# Rett syndrome: a review of clinical manifestations and therapeutic approaches

**DOI:** 10.3389/frsle.2024.1373489

**Published:** 2024-05-21

**Authors:** Katelyn Bricker, Bradley V. Vaughn

**Affiliations:** Department of Neurology, University of North Carolina School of Medicine, Chapel Hill, NC, United States

**Keywords:** Rett, neurodevelopment disorders, sleep disorder, epilepsy, developmental disabilities

## Abstract

Rett syndrome (RTT) is a severe X-linked dominant neurodevelopmental disorder predominantly affecting females and over 90% of these patients mutations linked to the methyl-CpG-binding protein 2 (MeCP2) gene. Although the syndrome is well noted for the classic repetitive hand motion with decline in speech, patients may have a wide range of cognitive and motor impairments. Typical comorbidities in RTT are characterized by poor growth, seizures, disrupted sleep, hyperventilation, breath holding spells, feeding difficulties, scoliosis, and behavioral issues. This paper aims to provide a brief overview of RTT, focusing on the clinical features of sleep and epilepsy, effects on childhood development, and available emerging treatment options. Sleep disturbances, epilepsy, and developmental regression can have profound effects on the quality of life in affected individuals. Current management strategies focus on a multidisciplinary approach to address symptoms and enhance overall wellbeing of individuals with RTT.

## 1 Introduction

Rett syndrome (RTT) is a severe neurodevelopmental disorder that primarily affects females, occurring in ~1 in every 10,000–15,000 live female births (Neul et al., 2022). RTT can occur in males but most will die before birth or in infancy. Over 90% of these patients mutations linked to the methyl-CpG-binding protein 2 (MeCP2) gene (Weaving et al., 2005; Zoghbi, 2005). The disease is characterized by loss of spoken language and development of hand stereotypies (Neul et al., 2010) in addition to other comorbidities affecting behavior, development, and motor function (Gold et al., 2018). This article provides an in-depth exploration of RTT, including its genetic basis, clinical manifestations, impact on sleep and epilepsy, childhood development, and available treatment options.

## 2 Genetic basis of Rett syndrome

RTT is predominantly caused by mutations in the Methyl-CpG-binding protein 2 (MECP2) gene located on the long arm of X chromosome (Xq28) (Amir et al., 1999). The MECP2 gene encodes an essential protein for neuronal function and the regulation of other genes. The MECP2 gene encodes a DNA methylation reader protein that functions in transcriptional repression, transcriptional activation, and RNA binding (Neul et al., 2008). Mutations can occur *de novo* or be inherited and lead to a loss of MECP2 function, having an effect on neuronal development and synaptic plasticity (Chahrour and Zoghbi, 2007). Approximately 90% of RTT patients have MECP2 mutations, a small portion carry mutations in other genes, such as CDKL5 and FOXG1, and can also result in phenotypes resembling RTT (Pejhan and Rastegar, 2021).

## 3 Clinical manifestations

### 3.1 Cognitive and motor impairments

RTT presents with a wide spectrum of clinical manifestations, with some of the most noticeable being cognitive and motor impairments. RTT has a typical course across four stages. Developmental regression begins at a young age and there is typically a period of deceleration of the rate of head growth. Initial development in affected girls is usually normal for the first 6–18 months, although subtle symptoms such as muscle hypotonia and deceleration of head growth are present earlier in life but not noticed until later. Then a period of developmental stagnation or regression occurs. Symptoms may not be prominent initially but can present over the first two clinical stages. Common symptoms include severe cognitive impairments, loss of purposeful hand skills, and the development of stereotypic hand movements, such as repetitive hand-wringing (Hagberg et al., 1983). A study comparing formal and informal methods for assessing language and cognition in RTT found that most children scored very low on standard expressive language assessment, only producing 1–3 word utterances and a spectrum of communicative functions through an eye gaze device (Ward et al., 2021). Some reports even demonstrate that RTT females can develop the potential for language communication. RTT patients exposed to 6 months of music therapy exhibited improved verbal/non-verbal communication skills, greater socialization skills, and more engagement in behavioral play (Chou et al., 2019). Although loss of spoken communication is common in this group, little is understood regarding receptive language function. Given the usual course is one of regression of function, we assume RTT girls acquire a basic level of language reception in early childhood. For example, when these children are systematically shown objects, pictures, or actions, they begin to laugh, become excited, or cry (Demeter, 2000). This basic receptive component of language is necessary for subsequent interventions to bolster communication (Sigafoos et al., 2023). Motor dysfunction in RTT includes gait abnormalities, muscle rigidity, and scoliosis. The loss of acquired motor skills, such as crawling or walking, is a feature of the disorder. Some studies have reported age-related motor deterioration among RTT. Clapping and mouthing of hands tend to be more prevalent in preschool ages, and this decreases in middle to late childhood (Carter et al., 2010). An analysis of 83 individuals with RTT found that the number of stereotypies decreased greatly after 10 years of age (Temudo et al., 2007). Research by Wong et al. (2019) reported that after the age of 10 years, the individual types or frequencies of hand stereotypic movements greatly decreased in 43 children with RTT.

A review of 32 studies reported the findings of movement disorders in RTT. Hand stereotypies were found to be almost universal, and diminished over time. The most notable movement disorder in RTT tends to be tremor. Gait disturbances with ataxia/tremor were common. Hypertonia often worsened with age, and dystonia/rigidity may also be present (Brunetti and Lumsden, 2020).

Scoliosis is frequently associated with this disorder, and the degree of scoliosis predicted the ability of patients to stand and to walk in a study on motor function in RTT (Rodocanachi Roidi et al., 2019).

#### 3.1.1 Childhood development

The developmental course of RTT is divided into four stages (Hagberg and Witt-Engerström, 1986):

1. Early stagnation: characterized by the loss of acquired skills and a plateau in development. Decreased head growth velocity is first noticed here. There is often less eye contact and social interaction, occurring between 6–18 months of age.
2. Rapid regression: marked by severe motor and cognitive decline, usually accompanied by hand-wringing and periodic breathing. Autistic features emerge. This stage occurs between age 1–4 years of age.
3. Pseudostationary phase: a period of relative stability, although individuals remain severely impaired. There may be some improvements in behavior and communication skills. Epilepsy is prominent in this stage. Occurs between age 2–10 years of age.
4. Late motor deterioration: a phase distinguished by progressive motor decline, muscle rigidity, and the development of scoliosis. Some will become wheelchair-dependent. Communication may improve but language is not regained. This stage often begins after age 10 years of age.

### 3.2 Communication and social interaction

Communication difficulties are prevalent in RTT, as affected individuals often lose their ability to speak and express themselves effectively. Social withdrawal, lack of eye contact, and impaired social interaction are characteristic features. RTT children have a considerable amount of difficulty shifting attention compared to typically developing children, measured with the use of eye tracking technology (Rose et al., 2019). There was also difficulty disengaging activity but this was to a lesser extent than shifting attention (Rose et al., 2019). Wang et al. (2022) found that younger children (≤ 10 years of age) with RTT have better socio-communicative skills in eye contact, alternate eye gaze, and turn-taking during social interaction procedures compared to their older counterparts (11–18 years).

## 4 Effects on sleep

Sleep disturbances affect approximately 80% of individuals with RTT (Didden et al., 2002). In a metanalysis of 19 studies Spruyt found most subjects had problematic sleeping, in particular, with 67% having complaints of excessive somnolence, 61% had difficulties initiating and maintaining sleep and 57% noted disturbed sleep not otherwise specified. More interestingly in a subgroup of females with MECP2-RTT demonstrated a higher prevalence rate of excessive somnolence and sleep-wake transition disorders than those diagnosed by CDKL5-RTT (Spruyt, 2022). Sleep disruption appears to occur in predominantly three categories: difficulty with sleep initiation and maintenance associated with increased daytime sleep, nocturnal events and sleep related breathing disorders. For many subjects, nocturnal sleep initiation is more difficult during the childhood to adolescence age range followed by a greater amount of waking after sleep onset. This nocturnal sleep disruption is also associated with an increase in daytime sleepiness and sleep suggesting an underlying circadian rhythm issue with some obtaining a biphasic sleep pattern and may explain some of the features found in formal overnight sleep studies. A study on sleep macrostructure in 21 RTT females found longer duration of wake time after sleep onset and total sleep time, higher stage N3, with lower stage N2 and REM (Zhang et al., 2023). In addition, nocturnal sleep disruption and circadian dysfunction, other autonomic nervous system dysfunction were especially associated with early truncating mutations indicating the widespread function of the MECP2 protein in the hypothalamus (Carroll et al., 2020). Some investigators have hypothesized that this nocturnal sleep and circadian disruption is related to a glutamate dysfunction (Johnston et al., 2015; Tascini et al., 2022). Caregivers also note a higher prevalence of nocturnal events. These events can include nocturnal laughter, nocturnal screaming and crying, sleep terrors, bruxism and nocturnal seizures. These findings mirror the results of a large sample study of 237 cases of Rett syndrome database, in which patients were noted to experience laughter during the night, daytime naps, night screaming, bruxism, seizures, obstructive sleep apnea, delayed sleep onset, and reduced sleep efficiency (Young et al., 2007). The most frequent noted is the nocturnal laughter. Complaints of nocturnal laughter and screaming appear to improve with time, and nocturnal laughter may be more common in those with larger genetic deletions (Wong et al., 2015). Parasomnia behavior of sleep terrors and sleepwalking is also noted with a greater prevalence of sleep talking (Spruyt, 2022).

Studies examining the prevalence of sleep related breathing have been mixed. In a sample of 11 subjects with RTT, more than half of the females with MECP2 mutations presented with obstructive sleep apnea in both NREM and REM sleep, unrelated to their clinical features. The subjects also presented with hypoxemia throughout nocturnal sleep (Zhang et al., 2022). A cohort of 13 patients with RTT were evaluated by polysomnogram and determined that sleep disordered breathing was prevalent in this cohort, with obstructive sleep apnea occurring most frequently (Sarber et al., 2019). However, Amaddeo in a study of 17 girls with RTT found that obstructive sleep apnea was only present when the child had adenotonsillar hypertrophy (Amaddeo et al., 2019).

Seizure severity and frequency appear to influence sleep quality. Various abnormalities have been described in the EEG during sleep including increased spike frequency (Aldrich et al., 1990), large-amplitude slow waves (Garofalo et al., 1988), and progressive background slowing suggesting changes in sleep regulation (Glaze et al., 1986). Nocturnal epileptic seizures appear to increase in frequency in late childhood and early adolescence. These seizures may be subtle focal onset or include more generalized seizures which may lead to missed events at night (Glaze et al., 1998). Sleep dysfunction and epilepsy may be linked. Boban et al. (2016) found that patients with more severe epilepsy reported greater sleep disruption. The sleep disruption can be severe as noted in some RTT patients developing electrical status epilepticus during sleep (Nissenkorn et al., 2010). This relationship is of greater sleep disruption correlated to more severe epilepsy is seen in other forms of epilepsy (Proost et al., 2022). Similarly, sleep deprivation and disturbed sleep are noted to increase the frequency of recurrent seizures but this is not studied in RTT patients. While the effect of improving sleep on the epilepsy and development appears to hold promise in other disorders, this relationship of epilepsy to sleep is unclear in patients with RTT.

## 5 Epilepsy in Rett syndrome

Epilepsy burdens a large portion of RTT individuals, with ~60%−80% experiencing seizures throughout their lifespan (Nissenkorn et al., 2010). In a study of 602 individuals with RTT, 48% of them were found to have seizures (Glaze et al., 2010). Epileptic seizures typically begin after age 3 years and increase with late childhood and puberty, but Nissenkorn found in their cohort 6% developed epilepsy in the first year of life, 43% in age 1–5 years and 20% after age 5 years (Glaze et al., 2010; Nissenkorn et al., 2010). Seizure onset before age 3 and after age 20 is uncommon, but earlier onset of seizures is associated with more severe phenotypes (Guerrini and Parrini, 2012; Nissenkorn et al., 2015). Types of seizures can vary but often include focal onset, tonic, atonic, myoclonic, and focal seizures. Seizure frequency often correlates with the severity of the RT syndrome but relationship to the degree or type of mutation is not clear. Patients with the CDKL5 and FOXG1 mutations are more likely to have a developmental epileptic encephalopathy. These seizures are often intractable afflicting over two thirds of patients of patients across all age spans (Henriksen et al., 2018). Although a minority of patients (< 15%) may never have seizures, only one in five of those with epilepsy may obtain seizure control. Epilepsy management in RTT may require antiseizure medications tailored to the specific seizure type and individual patient.

Electroencephalography typically shows a normal or slowing in the occipital region early in the course of the disorder. As the child progresses to Stage II, further background slowing and the development of epileptiform activity is noted. Typically these spikes are focal and then become more multifocal and generalized. Also with time, NREM sleep figures (K complexes and sleep spindles) become less distinct. By clinical stage III, rhythmical delta activity is noted in wake and sleep. In the later stage of the disorder marked background slowing, delta frequency rhythms and multifocal and generalized epileptiform activity is noted in both the awake and sleep states (Glaze et al., 1998).

RTT patients frequently have many types of behavioral and epileptic events that can be difficult to distinguish. Events may include recurring hand wringing, hyperventilation, breath holding, laughing, screaming, staring and unusual movements. These events can be mistaken for epileptic seizures if not critically evaluated using time locked video EEG. Although surface EEG may not capture the electrographic correlate to the event, further behavioral clues and reviewing multiple events can improve the correct identification, and may also reveal other behaviors that are epileptic (Glaze et al., 1998).

## 6 Treatment options and management

Management of RTT primarily focuses on symptomatic treatment, improving quality of life, and addressing comorbidities, such as sleep disturbances and epilepsy (Tarquinio et al., 2017).

### 6.1 Symptomatic treatment

- Physical therapy: to maintain mobility and reduce the risk of contractures.
- Occupational therapy: to improve fine motor skills and promote independence.
- Speech and communication therapy: to facilitate communication.
- Behavioral therapy: to manage challenging behaviors and improve social interaction.

### 6.2 Sleep disturbance management

Managing sleep disturbances in RTT may involve behavior modification, medication, and addressing potential underlying causes such as gastroesophageal reflux (Motil et al., 2012). First line therapy is sleep hygiene, including increasing daytime activities and light to decrease daytime sleeping, improvement of bedtime routines and sleep inducing environments. Some patients appear to have a better subjective quality of sleep with melatonin. A study conducted by Boban et al. evaluated 364 families with a child with RTT. It was found that of these patients, 274 were not taking therapy, 7.7% were on melatonin, 3.9% on clonidine, 3.3 on trazodone and 4.1 on other therapies (clonazepam, chloral hydrate, diazepam, oxazepam, antiepileptics, hypnotics e.g., zolpidem, and others). However, sleep hygiene strategies were the first line treatment option for ⅔ of the families and were deemed beneficial (Boban et al., 2018). Sleep medications did not substantially reduce sleep problems especially with combination treatment, as these individuals had more difficulty initiating and maintaining sleep (Boban et al., 2018). If behavioral interventions are not enough to improve sleep, then the most used drugs include melatonin, dopamine agonists and GABA agonists (Boban et al., 2018).

### 6.3 Epilepsy management

Individuals with RTT who experience seizures should be managed by neurologists with expertise in epilepsy. Treatment typically involves antiepileptic drugs tailored to the specific seizure types. Across several databases, carbamazepine appears to be most commonly used, with lamotrigine levetiracetam and topiramate cited as frequently used (Krajnc, 2015). Only small numbers of patients have been reported in studies suggesting effectiveness and thus head-to-head comparisons or larger double blind controlled trials are lacking. Newer treatment options are currently being researched. A study of five female children with drug-resistant epilepsy and pathogenic MECP2 mutation found that a dose of 10 mg/kg/day of cannabidivarin (CBDV) is safe and well tolerated in a pediatric RTT cohort and suggested improved seizure control in females with MECP2-related RTT (Hurley et al., 2022). Another investigation had a cohort of 26 RTT females with epilepsy, 10 patients were treated with cannabidiol (CBD) and seven out of 10 these patients had a >50% reduction in their reported seizures (Desnous et al., 2024).

### 6.4 Cognitive enhancement and neuroplasticity

Cognitive enhancement strategies are important in improving the overall functional outcomes in RTT individuals. Fabio et al. assessed the effect of cognitive training on behaviors and brain activity in 34 girls with RTT. The girls were divided into training groups that underwent long-term training and a control group (that did not undergo long-term training). Gaze and quantitative EEG data were evaluated in this study. The participants in long-term training looked faster and longer at the target, and showed increased beta activity and decreased theta activity, with a leftward asymmetry. While in the short-term training group, participants showed a habituation effect, decreased beta activity, and increased right asymmetry. Overall this study suggests a potential positive benefit from long-term cognitive training on brain and behavioral factors in RTT and may have inference for other communicative devices in these patients (Fabio et al., 2016). It is unclear, however, as to whether these findings translate to better cognition or spontaneous behavioral change.

Another study by Fabio et al. examined the neurophysiological and cognitive effects of transcranial direct current stimulation (tDCS) in three girls with RTT and chronic language impairments. tDCS stimulation was applied to Broca's area in addition to linguistic training. The results showed an increase in the number of vowel/consonant sounds and words and the production of comprehension through discrimination, improved motor coordination, and improved neurophysiological parameters (increased frequency and power of alpha, beta, and theta bands) (Fabio et al., 2018). Although the clinical implications of these findings are uncertain, this may provide a clue for further research into nonpharmacological therapies.

### 6.5 Medication management

In March of 2023, trofinetide was FDA approved for the treatment of RTT. Trofinetide is a novel synthetic analog of glycine-proline-glutamate (GPE), the N-terminal tripeptide of insulin-like growth factor 1 (Tropea et al., 2009). GPE naturally occurs in the brain and partially reverses the core symptoms in Mecp2-deficient mice, with improvements in respiratory function and heart rate, increasing brain weight, and prolonged life span (Tropea et al., 2009). The medication is thought to target specific pathways affected by MECP2 mutations by improving cognitive and motor function, reducing behavioral issues, and addressing sleep disturbance. Clinical trials demonstrated encouraging results leading to its FDA approval. The Lavendar study, a phase 3 clinical trial, assessed 187 females with RTT who took trofinetide or placebo. After 12 weeks of treatment, females who received trofinetide had greater improvements in their symptoms than those who took placebo. Participants who took trofinetide also communicated better than the placebo group. The most common side effects were mild-moderate diarrhea and vomiting (Neul et al., 2022).

## 7 Limitations

This review may be limited by potential biases such as publication bias or selective reporting bias, which may influence the interpretation of findings and final recommendations.

## 8 Conclusion

Rett syndrome is a complex and unique neurodevelopmental disorder primarily caused by mutations in the MECP2 gene. Its clinical manifestations, including cognitive and motor impairments, communication difficulties, sleep disturbances, and epilepsy, present remarkable challenges to affected individuals and their families. Clinical trials show promising evidence for treatment options that may symptomatically improve individuals with RTT. Novel medications such as trofenitide are showing promising effects in the improvement of cognitive and motor function, reduction of behavioral issues, and improvement in sleep disturbances. Additionally, cognitive enhancement strategies and transcranial direct current stimulation may help with behavioral factors, speech production, and comprehension in RTT. Although there is no cure for RTT, a multidisciplinary approach involving various therapies and management strategies aims to enhance the quality of life and attenuate the symptoms of individuals living with this disorder.

## Author contributions

KB: Writing – review & editing, Writing – original draft. BV: Writing – review & editing, Writing – original draft.
